# Hybrid PCA-Based and Machine Learning Approaches for Signal-Based Interference Detection and Anomaly Classification Under Synthetic Data Conditions

**DOI:** 10.3390/s25247585

**Published:** 2025-12-14

**Authors:** Sebastián Čikovský, Patrik Šváb, Peter Hanák

**Affiliations:** Department of Air Traffic Management, Faculty of Aeronautics, Technical University of Kosice, 04001 Kosice, Slovakia; patrik.svab@tuke.sk (P.Š.); peter.hanak@tuke.sk (P.H.)

**Keywords:** anomaly detection, sensor fusion, spatiotemporal data, low false positive rate, Neyman–Pearson thresholding, principal component analysis, local outlier factor, uncertainty estimation, Monte Carlo variance, distribution shift robustness

## Abstract

**Highlights:**

**What are the main findings?**

- A lightweight, interpretable three-signal fusion (PCA-MSE, LOF on residuals, MC-style variance) outperforms single detectors at a strict 1% FPR; on a 0 dB hold-out it lifts TPR@1% FPR from ~0.60 (baseline) to ~0.74.
- The fused detector is robust to level/SNR shifts (−12 to +12 dB), maintaining higher recall than baselines under degraded conditions.

**What are the implications of the main findings?**

- The NumPy/scikit-learn pipeline is easy to deploy on edge/embedded systems and transparent for auditing, thanks to train-only normalization and strict hold-out calibration.
- Neyman–Pearson–targeted thresholds support safety-critical sensing (e.g., industrial, medical, infrastructure) where false alarms must be tightly bounded while preserving sensitivity.

**Abstract:**

This article addresses anomaly detection in multichannel spatiotemporal data under strict low-false-alarm constraints (e.g., 1% False Positive Rate, FPR), a requirement essential for safety-critical applications such as signal interference monitoring in sensor networks. We introduce a lightweight, interpretable pipeline that deliberately avoids deep learning dependencies, implemented solely in NumPy and scikit-learn. The core innovation lies in fusing three complementary anomaly signals in an ensemble: (i) Principal Component Analysis (PCA) Reconstruction Error (MSE) to capture global structure deviations, (ii) Local Outlier Factor (LOF) on residual maps to detect local rarity, and (iii) Monte Carlo Variance as a measure of epistemic uncertainty in model predictions. These signals are combined via learned logistic regression (F*) and specialized Neyman–Pearson optimized fusion (F** and F***) to rigorously enforce bounded false alarms. Evaluated on synthetic benchmarks that simulate realistic anomalies and extensive SNR shifts (±12 dB), the fusion approach demonstrates exceptional robustness. While the best single baseline (MC-variance) achieves a True Positive Rate (TPR) of ≈0.60 at 1% FPR on the 0 dB hold-out, the fusion significantly raises this to ≈0.74 (F**), avoiding the performance collapse of baselines under degraded SNR (maintaining ≈ 0.62 TPR at −12 dB). This deployable solution provides a transparent, edge-ready anomaly detection capability that is highly effective at operating points critical for reliable monitoring in dynamic environments.

## 1. Introduction

The ability to reliably detect and interpret anomalies in multidimensional data streams has been a fundamental challenge in signal analysis, cyber-physical monitoring, and intelligent fault diagnosis in recent years [1,2]. In contemporary sensor networks and communication systems, interference and abnormal signal patterns frequently develop in complex, nonlinear, and correlated feature spaces. Classic statistical methods like Principal Component Analysis (PCA) or spectral estimation are still useful for dimensionality reduction and visualization but are unable to resolve in-depth and context-dependent anomalies in dynamic environments [3,4,5]. To improve diagnostic precision, in the past years more and more research has been concerned with hybrid detection frameworks combining reconstruction-based learning, probabilistic reasoning, and local density estimation [6]. These strategies combine model-derived and data-driven behavior principles with the aim to enhance interpretability and robustness in heterogeneous signal environments. Fundamental to anomaly detection is the ability to measure the deviations of the model from the nominal system. Reconstruction-based approaches, such as PCA, autoencoders, and probabilistic extensions, project data onto a lower dimensional subspace and determine the mean squared error (MSE) between the original and reconstructed data. When trained on normal data, large reconstruction errors indicate deviations from an expected structure [3,4,7]. Classical PCA also has to assume linear correlations and Gaussian noise; although it is much simpler and lower computational cost, it can efficiently detect outlying patterns in industrial and communication data samples.

The probabilistic approach to PCA furthered its theoretical grounding by correlating PCA with maximum-likelihood inference, in which latent variables and covariance relations can be directly estimated from the data [3]. Robust subspace formulations extend this logic into Robust PCA, one able to decompose matrices into low-rank and sparse components, defending from gross noise and partial corruption commonly found in spectrogram or sensor samples [4]. Drawing on these foundations in this work, Takeishi (2019) described an interpretable extension for anomaly detection based on PCA by calculating Shapley values of reconstruction errors [7]. This method assesses the contribution of all features on the total deviation, addressing the obscurity of the dependent variable causing indirect propagation of error. By introducing a probabilistic value function into the PCA model, this approach could better represent the anomalies across the features and keep strict mathematical consistency. Such explainable detection conforms to the current trend of transparency in industrial machine learning, particularly in safety-sensitive applications where each decision must be traceable [8]. Apart from ‘pure subspace’ methods, hybrid anomaly detection can bring to bear more emphasis its combination of model interpretability and deep learning adaptiveness.

Puder et al. (2024) integrated Kalman filtering and neural networks within a hybrid methodology for time series anomaly detection [8]. The Kalman element captures linear temporal dynamics and noise statistics, and deep embedding helps to adjust residual patterns using nonlinear mappings. Our hybrid structure enhances the stability of the concept drift-based prediction and detection sensitivity when the data have noise or insufficient information. Other related work has proposed a deep embedding optimized Kalman filter called KalmanAE that combines reconstruction loss with latent state estimation [9]. These hybrid approaches portray a transition from static statistical models to those for a composite system that combines physical insight and data-driven optimization. Parallel developments can be seen in local density-based anomaly detection. The Local Outlier Factor (LOF) algorithm of Breunig et al., 2000 and its probabilistic successor LoOP (Kriegel et al., 2009) measures how much the local deviation of your object is relative to its neighbors in feature space [10,11]. Unlike global distance measures, however, these methods are sensitive to the size and density of the data being studied, detecting context-dependent outliers in heterogeneous clusters.

Campos et al. (2016) systematically tested LOF and additional algorithms through different datasets: the emphasis is that no one method prevails over all conditions but to benefit of more optimal generalizability it is best to combine local density measures with some reduction or prediction error models [12]. Subsequent research (Hamlet and Kinsner 2017; Alghushairy et al. (2021), applied LOF to both data streams and large-scale computing contexts, which allows for real-time detection in sensors and communication systems [13,14]. Local density descriptors thus make it a complement to reconstruction-based hybrid architectures, which enable them to enhance thresholds and adaptively quantify anomaly confidence. In the past few years, signal-based interference detection has become an important practical area to test these theoretical advances. The increasing density of radio and satellite communication systems has rendered interference a crucial performance limitation. Morales Ferre et al. (2019) applied machine learning classifiers on spectrogram-based representations of Global Navigation Satellite System (GNSS) signals to spot and classify jamming patterns [15]. Their results confirmed that time–frequency representations offer rich discriminative features that allow convolutional networks and ensemble approaches to distinguish between unintentional noise, deliberate jamming, and multipath interference.

Rijnsdorp et al. (2023) also proved how spectral power density maps and learned embeddings support real-time interference classification on embedded devices [16]. Similarly, convolutional architectures trained on spectrograms can capture jamming signals accurately, showing that deep learning can exploit the spatial continuity of the frequency–time energy patterns [17,18,19]. These reports show that the transformation of raw waveforms into two-dimensional spectrograms remains one of the most powerful methods of preprocessing for anomaly recognition in radio and acoustic fields. In such frameworks, error metrics play a significant role in quantifying model performance. The Mean Squared Error is still the basic reconstruction metric in PCA and autoencoder systems because of its simplicity and analytical tractability [3,4,19]. Yet, due to its weakness in the case of imbalanced or noisy datasets, it is encouraged to favor more informative statistical measures. Saito and Rehmsmeier (2015) have shown that the precision–recall curve is a better comparison than ROC in imbalanced classification problems [19]. These works also emphasize the importance of careful metrics that take into account an understanding of both sensitivity and specificity in their assessment of anomaly detectors working on rare event datasets [19,20,21]. Uncertainty quantification has also turned into a vital part of contemporary hybrid detection systems.

Gal and Ghahramani (2016) interpreted dropout regularization in neural networks as a Bayesian approximation, revealing that repeated stochastic dropout sampling gives Monte Carlo (MC) estimates of predictive variance [22]. This principle affords hybrid models the ability to represent epistemic uncertainty—how confident the model is about its prediction—which is essential for working systems within communication and navigation. Choubineh et al. (2023) refined more MC dropout techniques for uncertainty measurement in convolutional architectures, showcasing that uncertainty estimates have the potential to enhance calibration and anomaly confidence assessment without retraining [23]. The joint method that combines this reconstruction error magnitude with MC variance serves as a two-dimensional criterion that discriminates between high error regions in the form of noise and genuine anomalous regions, making robust hybrid decision rules feasible.

The unification of these research paths points toward a new iteration to detect anomalies based on complementary concepts. PCA and its probabilistic variants still provide efficient subspace representations that accommodate prominent correlations, whereas hybrid Kalman deep embeddings add temporal adaptiveness and noise resilience [9,24,25]. Local density approaches such as LOF and LoOP contribute contextual sensitivity, and Bayesian dropout techniques quantify uncertainty [10,11]. If the signal is converted into the spectral–temporal domain for spectrogram-based interference detection, the approach can be formulated as a coherent methodology in which features are reduced by PCA reconstruction, anomalies are identified using MSE and variance metrics, and correlations are confirmed using local density measures and probabilistic confidence estimation [3,4,5,15,16,17,18,22,23]. This view is integrated with recent state-of-the-art signal processing and intelligent diagnostics which has similarly prioritized explainability, adaptability, and statistical rigor. Reconstruction-based detection provides the structural foundation while hybrid learning guarantees adaptability based on nonstationary data and density and uncertainty models confirm interpretability. Such hybrid models and explainable methods provide high sensitivity in complex interference situations while also preserving computational cost-effectiveness that is suitable for real-time applications, as shown in the reviewed works [15,16,17,18]. In the sections below, the paper elaborates on these concepts to introduce and experimentally demonstrate a hybrid PCA-based approach to interference identification through spectrogram analysis and advanced evaluation metrics.

Hybrid architectures such as Kalman–deep embedding models combine linear state estimation with neural-network-based representation learning, but they typically rely on a single global residual signal and lack explicit mechanisms for uncertainty quantification, which limits their ability to operate at strict low-FPR thresholds under distribution shift.

Pure PCA-based detectors also provide only global reconstruction deviation and assume linear correlations and stationary noise, making them insensitive to localized or structurally rare anomalies that appear within residual maps.

The proposed approach explicitly overcomes these limitations by integrating three complementary anomaly signals: (i) global structure deviation via PCA-ME, (ii) local rarity detection via LOF on residual maps, and (iii) epistemic uncertainty estimation via Monte Carlo variance.

This triad enables the detector to capture anomalies that are global, local, or uncertainty-driven, and supports Neyman–Pearson thresholding that maintains 1% FPR even under SNR shifts. As demonstrated in Section 4, the fused detector achieves substantially higher TPR@1% FPR than all single baselines, including both PCA-only and uncertainty-only models. ENF-based interference monitoring in distributed sensor networks is subject to several structural constraints that directly motivate the need for a fused anomaly detection approach. First, spatiotemporal correlations in ENF signals mean that disturbances may be globally coherent across sensors but locally expressed in only small regions of the time–frequency representation, making purely global metrics insufficient. Second, real-world sensor nodes often experience heterogeneous and time-varying SNR conditions, resulting in inconsistent reconstruction difficulty and unstable threshold behavior when using a single detection metric. Third, interference in practical environments is frequently non-stationary, with short transients, narrowband injections, or localized bursts that cannot be reliably captured by global reconstruction error alone. Finally, ENF-based monitoring systems typically require strict false-alarm control (e.g., 1% FPR), because false detections trigger network-level actions or system alarms.

These challenges motivate the proposed fusion of PCA-based global reconstruction deviation, LOF-based local rarity detection in residual maps, and MC-derived epistemic uncertainty, which together allow the detector to remain stable under SNR variation and sensitive to both global and localized anomalies.

## 2. Methodology

This study formalizes the anomaly score definitions, fusion strategies, and evaluation protocol for robust anomaly detection in multichannel spatiotemporal data. All definitions and thresholds are calibrated exclusively on a validation subset under a strict hold-out regime, ensuring that test data are never used in threshold or weight learning [3]. All experiments were implemented in Python 3 using NumPy, SciPy, and scikit-learn. The autoencoder was trained with a standard deep learning library supporting dropout and Adam optimization. Figures were produced with Matplotlib (version 3.10.0.), with random seeds fixed to ensure reproducibility.

### 2.1. Anomaly Scores

Let **x** ∈ R^H×W^ denote one sample, flattened into **x** ∈ R^d^ with d = HW. Each baseline method returns a scalar score s(**x**), which is later thresholded into a binary prediction ŷ ∈ {0,1}.

#### 2.1.1. PCA Reconstruction MSE (B1)

After centering the data, we project onto the first k principal components **U**_k_ ∈ R^d×k^ and reconstruct $\hat{x}$ = **U**_k_ **U**_k_^T^ **x**. The anomaly score is the mean squared reconstruction error [4]:

(1) $$s_{\mathrm{mse}}\left(x\right)=\frac{1}{d}||x-\hat{x}{\left|\right|}_{2}^{2}\;=\frac{1}{d}\;||I\;-\;U_{k}U_{k}^{⊤}x{\left|\right|}_{2}^{2}.$$

where **x** ∈ R^d^ is the centered input and **U**_k_ ∈ R^d×k^ contains the first k– principal components d = HW.

#### 2.1.2. LOF on the Error Map (B2)

From a reconstructor (linear or autoencoder), we obtain an error map e(**x**) ∈ R^H×W^. After vectorization z = vec(e(**x**)) ∈ R^d^ we compute the Local Outlier Factor. For k-nearest neighbors, the local reachability density and LOF are given by [10]:

(2) $${\mathrm{reach}-\mathrm{dist}}_{k}\left(p,o\right)=max\{k-\mathrm{dist}\left(o\right),{\left\|p-o\right\|}_{2}\},$$

(3) $${\mathrm{lrd}}_{k}(p)={(\frac{1}{\left|N_{k}(p)\right|}\sum_{o\in N_{k}(p)}{\mathrm{reach}-\mathrm{dist}}_{k}(p,o))}^{-1},\;$$

and score

(4) $$s_{\mathrm{lof}}\left(p\right)={\mathrm{LOF}}_{k}\left(p\right)=\frac{1}{\left|N_{k}\left(p\right)\right|}\sum_{o\in N_{k}\left(p\right)}\frac{{lrd}_{k}\left(o\right)}{{lrd}_{k}\left(p\right)}.$$

#### 2.1.3. Monte Carlo Variance (B3)

For T stochastic forward passes of a model with dropout, we obtain partial scores a_t_(**x**) (e.g., MSE at the output). The empirical mean and variance [22]:

(5) $$z(u)=\frac{u-\mu_{C}}{\sigma_{C}},\;s_{mse+lof}(x)=\frac{1}{2}(z(s_{mse}(x))+z(s_{lof}(x)))$$

where μ_C_ and σ_C_ denote the mean and standard deviation on C.

To obtain the Monte Carlo variance score used as the third baseline component (B3), we compute T stochastic forward passes producing partial scores a_t_(**x**) (e.g., reconstruction MSE). The MC-variance anomaly score is defined as:

(6) $$s_{\mathrm{mc}}\left(x\right)=\frac{1}{T-1}\sum_{t=1}^{T}{\left(a_{t}\left(x\right)-\bar{a}\left(x\right)\right)}^{2},$$

where

(7) $$\bar{a}\left(x\right)=\frac{1}{T}\sum_{t=1}^{T}a_{t}\left(x\right).$$

This variance measures epistemic uncertainty and forms the third baseline score (B3) used later in the fusion models.

### 2.2. Hybrid Score Fusion

Three fusion variants are considered.

F* (learned fusion). We model the combination via logistic regression [25]:

(8) $$\begin{array}{rr} & s\left(x\right)=\left[\begin{array}{c} s_{mse}\left(x\right)\\ s_{lof}\left(x\right)\\ s_{mc}\left(x\right) \end{array}\right],p_{\theta }\left(y=1|x\right)=\sigma \left(w_{0}+w^{⊤}s\left(x\right)\right),\\ & L(\theta )=-\sum_{\left(x,y\right)\in C}\;\;\left[y\log p_{\theta }+\left(1-y\right)\log\left(1-p_{\theta }\right)\right]+\lambda \|w{\|}_{2}^{2}. \end{array}\;$$

using L2 regularization and binary cross-entropy optimization on a strictly disjoint calibration set C.

F** (Maximizing TPR under constrained FPR). We search for weights w ∈ Δ^2^ (simplex), such that the linear score [24,26]:

(9) $$s_{lin}\left(x\right)=w^{⊤}s\left(x\right),$$

(10) $$w\in \Delta^{2}=\left\{w\geq 0,1^{⊤}w=1\right\}.$$

achieves the highest TPR on C while maintaining FPR ≤ α (α = 1% in our case). Weights are selected by grid search, and the threshold is defined using the Neyman–Pearson criterion.

F*** (Neyman–Pearson Thresholding of F*). On the output of F*, we choose the threshold $\tau_{\alpha }$ such that ${FPR}_{C}(\tau_{\alpha })\leq \alpha$, and apply this fixed threshold to $D_{\mathrm{test}}$. The Neyman–Pearson lemma guarantees Type I error control under unknown distributions, provided the validation remains stable and information leak-free [26].

### 2.3. Calibration and Standardization

For each shift level ${\mathrm{shift}}_{\mathrm{dB}}$, a separate calibration subset C is constructed without any data leakage to the test set. The threshold for F1* is chosen as [26]:

(11) $$\tau^{⋆}=arg\;\underset{\tau }{max}F_{1}\left(\tau \right)\mathrm{on}\;C,$$

while the Neyman–Pearson threshold for α = 1% is defined analogously:

(12) τα=inf{τ:FP^RC(τ)≤α}.

These fixed thresholds are then applied to $D_{\mathrm{test}}$ under the same shift scenario.

### 2.4. Metrics and Evaluation Method

Given the score s(x) and threshold τ, classification is performed as ŷ=1{s(x)≥τ}  [1]. From the confusion matrix, we compute the basic measures (with TP, FP, TN, FN denoting its elements):

(13) $$\mathrm{Precision}=P=\frac{TP}{TP+FP},\;\mathrm{Recall}=R=\frac{TP}{TP+FN},\;FPR=\frac{FP}{FP+TN},$$

The F1 score for a given τ is

(14) $$F_{1}(\tau )=\frac{2PR}{P+R}.$$

We also report ROC AUC and PR AUC. In imbalanced datasets, PR AUC is usually more informative [6,7]. For a compact and imbalance-robust summary, we use the Matthews Correlation Coefficient (MCC) [20]:

(15) $$MCC=\frac{TP\cdot TN-FP\cdot FN}{\sqrt{\left(TP+FP)(TP+FN)(TN+FP)(TN+FN\right)}}$$

which has advantageous properties even under strong class imbalance. In applications with strict false-alarm limits, we explicitly report TPR at fixed FPR levels (e.g., TPR@1% FPR), directly corresponding to operational requirements [19,21].

### 2.5. Implementation Details

Preprocessing is uniform across all experiments, including centering and scaling according to the statistics computed from the training data. For PCA, the number of components k  is chosen based on the ratio of explained variance on $D_{\mathrm{val}}\;$ or fixed for comparability across methods [4].

The reconstruction model that generates error maps is trained exclusively on normal samples. During inference, dropout remains active to enable T-fold Monte Carlo (MC) estimates of epistemic uncertainty—typically several tens of forward passes as a practical compromise between variance and computational cost [22].

The LOF algorithm is applied using the Euclidean metric, with the number of neighbors k selected on the calibration subset C. The known sensitivity of LOF to this parameter is discussed in the original work [27]. The learned fusion method (F*) is implemented as a logistic regression with L2 regularization, optimized via binary cross-entropy on C. All thresholds ($F1^{*}$, $\tau_{\alpha }$) are determined exclusively on C and applied unchanged during testing to prevent any form of data leakage [26,27].

### 2.6. Model and Training Specification

The autoencoder component is realized using a linear autoencoder equivalent to Principal Component Analysis (PCA), trained exclusively on normal samples in a one-class configuration. A latent space of dimension k = 30 was selected. This choice was not based on maximizing the explained variance (which would require k ≈ 80), but rather on the principle of noise filtration. As demonstrated by the cumulative explained variance curve, k = 30 captures approximately 65% of the total variance. Components beyond this point are deemed primarily associated with noise modeling and high-frequency variations, which we explicitly seek to ignore to ensure a more robust and generalized representation of the nominal subspace. This approach prevents the model from reconstructing potential anomalies.

For the Monte Carlo (MC) Variance estimation, MC Dropout was employed, with the dropout probability set to *p* = 0.2 during both training and inference. The estimation of epistemic uncertainty was based on T = 20 independent forward passes.

For the Local Outlier Factor (LOF) computed over the reconstruction error map, we utilized the standard Euclidean metric. The number of neighbors k was optimized as a hyperparameter in the range of [10, 99] on the calibration set, typically converging to k ≈ 50.

Optimization for the autoencoder was performed using the Adam algorithm with an initial learning rate of 1 × 10^−3^, a batch size of 64, and early stopping based on validation reconstruction error with a patience of 10 epochs (maximum 200 epochs). All models were implemented in Python 3.9.13 using NumPy 1.21.0 and scikit-learn 1.0.2 to ensure reproducibility and computational efficiency for edge deployment.

A systematic sensitivity analysis over wider ranges of k and T is left for future work, as it would require substantially extending the experimental grid beyond the scope of this study.

### 2.7. Fusion Parameterization and Weight Interpretation

The learned fusion F* is a logistic regression with L2 regularization (λ = 1.0) trained on C over the triplet of scores s(x) = [$s_{\mathrm{mse}}$, $s_{\mathrm{lof}}$, $s_{\mathrm{mc}}$]^⊤^. Calibrated weights (intercept = −4.7530, $w_{\mathrm{mse}}\;$= 1.4512, $w_{\mathrm{lof}}$ = 1.2098, $w_{\mathrm{mc}}$ = 3.0101) demonstrate the dominance of the uncertainty component. In F**, weights are chosen by grid search on a simplex grid with step 0.02. For the NP mode, optimal weights are $w_{\mathrm{mse}}$ ≈ 0.00, $w_{\mathrm{lof}}$ ≈ 0.36, $w_{\mathrm{mc}}$ ≈ 0.64. In both cases, the individual component scores are standardized (z-score) according to C before combination to avoid dominance of any single metric.

**Figure 1:**
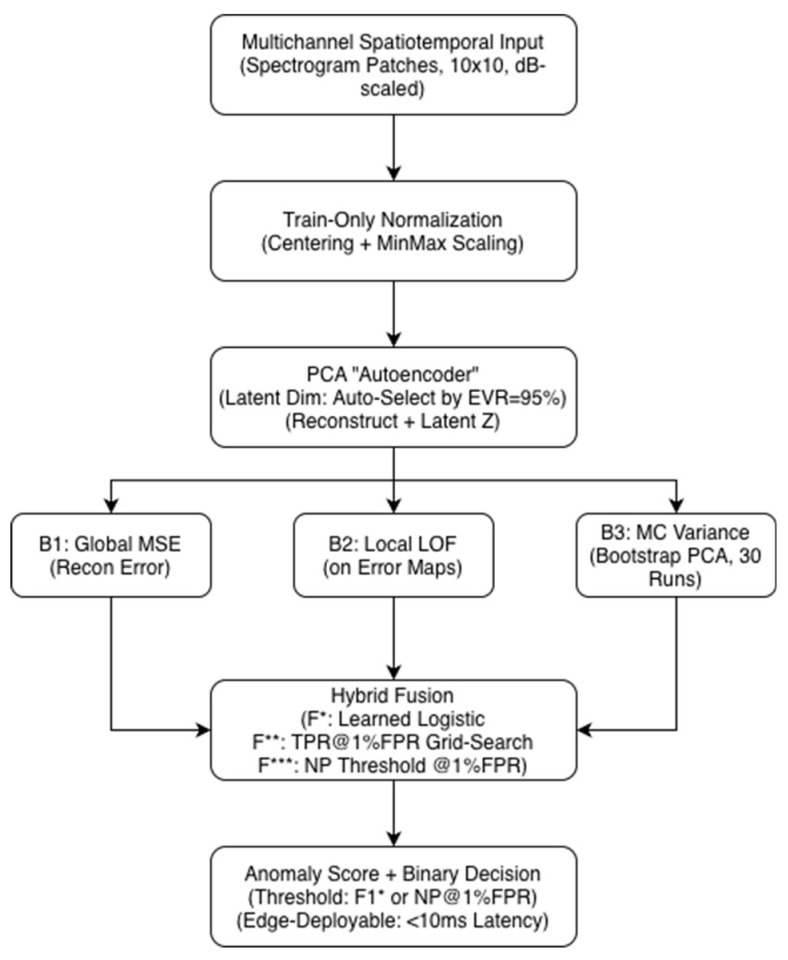
System architecture of the proposed anomaly detection pipeline.

The complete processing pipeline described in Section 2.1, Section 2.2, Section 2.3, Section 2.4, Section 2.5, Section 2.6 and Section 2.7 is summarized in Figure 1.

Figure 1 illustrates the full processing chain from multichannel spectrotemporal input through preprocessing, normalization, PCA-based reconstruction, Local Outlier Factor computation on residual error maps, and Monte Carlo variance estimation. The three baseline scores (B1: MSE, B2: LOF, B3: MC variance) are combined via hybrid fusion (F*, F**, F***) to produce a final anomaly score, which is thresholded using either the F1* or the Neyman–Pearson criterion for low-FPR operation. The figure also highlights the pipeline’s suitability for edge deployment with sub-10 ms latency.

## 3. Experimental Setup and Data Scenarios

In this section, we describe in detail the data, the synthetic simulation of distributional shift, the division into training/validation/test sets, the calibration protocol without information leakage, the selection of metrics, and the practical settings necessary for reproducibility. The chosen protocol is designed to separate (i) model and threshold tuning from (ii) final evaluation, while also enabling an analysis of robustness to controlled data shifts [22,26,28].

### 3.1. Data and Partitioning

We work with multichannel spatiotemporal samples of the form $x\in R^{H\times W}$, where in our experiments H=W=10. The entire corpus is divided into a clean training set $D_{\mathrm{train}}$ (320 samples), a clean validation set $D_{\mathrm{val}}$ (80 samples), and a test set $D_{\mathrm{test}}$ (500 samples), with the test set containing a mixture of normal and anomalous data. The contamination in the test set is approximately 5% (parameter contam = 0.05). Training and validation are performed exclusively on normal samples (one-class configuration), which is the standard procedure in unsupervised or semi-supervised anomaly detection and reduces the risk of information leakage [1,25].

The dataset in this study is not partitioned using a Dirichlet non-IID process, as the work does not involve federated or multi-client learning. All samples in D_train_, D_val_ and D_test_ are drawn i.i.d. from the same clean ENF-generating process, and the only controlled deviation is the additive SNR shift described in Section 3.2. A deterministic random seed is used for all sampling and split assignments to ensure full reproducibility. Because the data consist of ENF-based waveform signals and spectrogram-derived representations, no lighting or illumination-dependent variability is present in the acquisition process. Noise amplitude and interference type are the only varying factors.

### 3.2. Synthetic Simulation and Ditributional Shift

We assess robustness through a controlled additive noise shift with intensity shift_dB_ ∈ {−12, −6, 0, +6, +12} dB. Let

(16) $$n\sim N(0,\;\sigma^{2}I),$$

denote white noise. We define the modified sample as:

(17) $$x^{\prime }=x+\alpha (dB)n.$$

where the amplitude factor—that is, we scale the noise amplitude according to the usual dB convention. Each shift level is evaluated separately, and for it we construct a separate calibration subset C ⊂ D_val_ ∪ D_train_ (without any use of D_test_) for fusion training and threshold selection [26,28].

We introduce two evaluation regimes:

**(i) Full test.** We concisely report metrics on the entire D_test_ with fixed hyperparameters and thresholds from calibration. This view is useful for quick orientation but is not used for optimization [21,29].

**(ii) Strict hold-out (strict).** Calibration of the fusion (F*) and selection of thresholds (F1*, NP@1 %) is performed exclusively on C; thresholds are applied unchanged to D_test_. This regime explicitly controls Type I error under NP thresholding and eliminates data leakage [24,26].

The distributional shift is modeled by additive white noise n∼N (0, σ^2^I), scaled to the chosen amplitude. The noise is added at the input after standardization according to the training statistics and before projection to PCA or before AE processing. The required amplitude scaling factor α for the noise injection is calculated to simulate the signal-to-noise ratio (SNR) shift (in dB) using the relation: $\alpha (dB)=10^{\frac{{\mathrm{shift}}_{\mathrm{dB}}}{20}}$. For reproducibility we fix random seeds and list library versions and hardware. For each run we record the identifiers of samples in C and in D_test_ so that the split can be replicated exactly.

### 3.3. Evaluated Methods and Hyperparameters

Before spectrogram extraction, each raw signal undergoes a noise-robust preprocessing stage designed to suppress transient fluctuations unrelated to ENF dynamics. We employ a Gaussian Mixture Model (GMM)-based motion/noise detector, which models the short-term amplitude distribution using a mixture of low-variance (stationary) and high-variance (transient) components. Samples classified as transient or impulsive are attenuated, while stationary ENF-dominated segments are preserved. This procedure stabilizes the residual maps used in LOF and reduces variance in PCA reconstruction, which is crucial in low-SNR environments. The ENF signal and its harmonics lie almost entirely below 200 Hz, and higher-frequency components contain mainly noise rather than discriminative structure. Therefore, we downsample all signals to 1 kHz, which satisfies the Nyquist criterion for ENF analysis while providing three benefits: (i) a significant reduction in computational load for PCA reconstruction and MC sampling, (ii) lower variance in local residual maps used by LOF, and (iii) improved robustness to high-frequency sensor noise. Empirically, downsampling below 1 kHz reduced detection performance, while using higher rates did not yield additional accuracy but increased computational cost. Thus, 1 kHz is a robust and efficient compromise for edge-device deployment.

We evaluate four baseline scores and three fusion variants (as in Chap. 2): PCA-MSE (B1) [4], LOF on the error map (B2) [10], MC-dropout variance (B3) [22], combined MSE+LOF (B4) [10], and fusions F* (logistic regression) [25], F** (TPR optimization at FPR = 1%) [24,26], and F*** (NP thresholding of the F* output) [26]. For PCA we use k = 30. For LOF we select the number of neighbors k and the metric on C by maximizing MCC [20]. MC-dropout during inference computes multiple passes, and we interpret the empirical variance of the score as epistemic uncertainty [22]. The MSE+LOF combination is standardized component-wise (z-score) on C so that no single component’s scale dominates [10].

The learned fusion F* is modeled as logistic regression with L2 regularization, trained with binary cross-entropy on C with input s(x) = [$S_{\mathrm{mse}}$, $S_{\mathrm{lof}}$, $S_{\mathrm{mc}}$]^T^ [10]. F** searches for weights w of the linear combination w^T^s(x) that maximize TPR at FPR ≤ 1% (grid search on C), thereby realizing a practical version of Neyman–Pearson classification [22,24]. F*** applies Neyman–Pearson thresholding directly to the output of F*, i.e., selects τ_α_ such that FPRC (τ_α_) ≤ α for α = 1%, and then applies this threshold fixed in the test [22,24].

The PCA-equivalent autoencoder used in this work operates on 10 × 10 spectrogram patches with latent dimension k = 30, resulting in a total of 6130 trainable parameters. A single encoder–decoder pass requires approximately 4 kd ≈ 1.2 × 10^4^ operations, and the full Monte Carlo variance computation with T = 20 stochastic passes amount to roughly 2.4 × 10^5^ operations per sample. This computational budget is several orders of magnitude lower than CNN–Bi-GRU–Attention architectures typically used for audio analysis and fits well within the capabilities of low-power edge devices, even without GPU acceleration.

### 3.4. Threshold Calibration and Metrics

For each shift scenario and each method, we define two thresholds:

For F1* thresholding we choose τ* = argmax_τ_ F1(τ) on C. NP thresholding (NP@1%). We define τ_0.01_ as the lowest threshold at which the estimated FPR on C does not exceed 1%. This procedure explicitly controls Type I error [26,30]. The evaluation metrics include ROC AUC, PR AUC (in imbalanced tasks more informative than ROC) [19], F1, TPR@1% FPR, TPR@5% FPR, accuracy, and MCC (a robust correlation metric) [20].

### 3.5. Evaluation Protocol and Reporting

To keep results understandable and free of leakage, for each value of shift_dB_ we repeat the following procedure:

(1) prepare the shifted versions of C and D_test_;
(2) train F* on C and/or find weights for F**;
(3) determine τ* (F1*) and τ_0.01_ (NP@1%) on C;
(4) apply the thresholds to D_test_ without change and compute the metrics.

Finally, we report two tables: “Full test” and “Strict hold-out,” with the strict regime considered authoritative [26,28].

### 3.6. Statistical Analysis and Confidence Intervals

In addition to point estimates of the metrics, we report 95% bootstrap confidence intervals with 10,000 resamples at the sample level. For comparing ROC AUC, we provide DeLong’s test. For PR AUC we use a paired bootstrap. For TPR@1% FPR we also report a confidence interval for TPR derived by bootstrap over the pairs (TP, FP).

### 3.7. Implementation and Practical Notes

The baseline methods are implemented with standard libraries. LOF uses the Euclidean metric with the number of neighbors k selected on C (typically in the tens), and we standardize the score if it enters a fusion [10,25]. Dropout is active during inference and the variance is computed from multiple passes (practically “tens” of repetitions), which is a proven way to estimate epistemic uncertainty [22]. All thresholds and weights are tied to the specific shift_dB_ scenario and are not transferred across scenarios. This yields a clean attribution of the shift’s impact on the detector rather than on the calibration [28].

## 4. Results

This chapter summarizes the detection performance of the proposed methods and their fusions. First, we analyze the primary scenario at 0 dB on the strict hold-out set without information leakage, then we evaluate robustness to level shifts (−12, −6, 0, +6, +12 dB) and discuss the contribution of the individual fusion components (MSE, LOF, MC-variance). Finally, we discuss operational thresholds and the difference between the “full test” and the strict hold-out.

### 4.1. Performance at 0 dB (Strict Hold-Out)

The baseline comparison framework is the strict hold-out at 0 dB. Complete values are given in Table 1. The best single method is MC-variance (B3) with ROC AUC ≈ 0.9235, PR AUC ≈ 0.8517 and F1* ≈ 0.7674, reaching TPR@1%FPR ≈ 0.60. The learned logistic fusion F* improves PR AUC to ≈ 0.8861 and F1* to ≈0.8539 at TPR@1%FPR ≈ 0.72. The fusion optimized for low error rate F** achieves the highest ROC AUC ≈ 0.9343, F1* ≈ 0.8571 and at the same time shifts TPR@1%FPR to ≈ 0.74 (higher than all baselines). These values indicate that combining MSE + LOF + MC through learned/optimized weights provides a significant benefit under strict false-alarm limitations.

**Figure 2:**
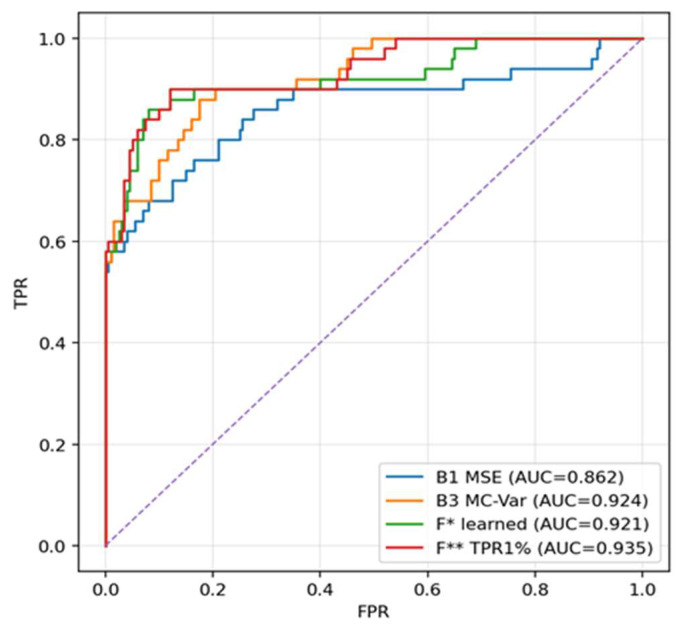
ROC curve, 0 dB, strict hold-out.

**Figure 3:**
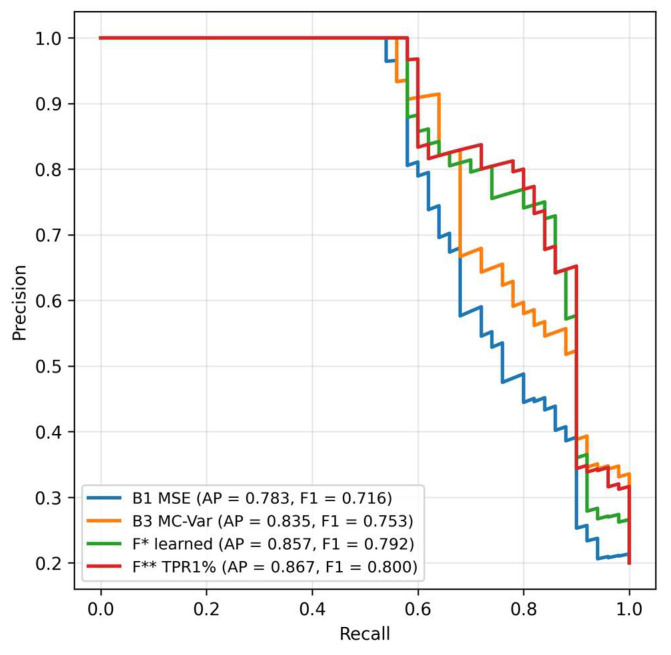
Precision–recall curves, 0 dB, strict hold-out.

As can be seen from Table 1, on the held-out hold-out B3 (MC-variance) achieves very good AUC metrics, but the fusion (F*, F**) outperforms it mainly in the region of low FPR, which is visible on TPR@1%FPR and in the shape of the curves in Figure 2 and Figure 3. The learned logistic fusion F* increases PR AUC and F1* while maintaining high detection at 1% FPR, whereas F** intentionally maximizes TPR at FPR ≈ 1% by further threshold adjustment. This table is decisive in the article for statements about generalization, as it separates the calibration phase from the evaluation.

Comparison of ROC curves (B1–B4 vs. F, F*, F***) at 0 dB. The purple diagonal line represents the performance of a random classifier (the line of no-discrimination), serving as the minimum acceptable benchmark (AUC = 0.5). The curves show that the fusions (especially F** optimized for TPR@1%FPR) exhibit a steeper rise on the left and reach higher TPR at very low FPR than the individual baseline methods. MC-variance (B3) is the strongest single method, but in the region of FPR ≈ 1% the fusion surpasses it. This ROC shape is important for applications that must keep false positives to a minimum.

In the precision–recall space the fusions maintain higher precision with increasing recall, meaning fewer false alarms at comparable detection. The difference is most pronounced in the middle part of the recall range, where LOF complements MSE and MC-variance and together they keep higher reliability of positive findings without a drastic drop in sensitivity (Figure 2).

Precision–recall curves at 0 dB. The fusions maintain higher precision at medium and higher recall values. PR AUC is highest for F* and F**. Figure 3 confirms that F* achieves PR AUC ≈ 0.8568 and F** ≈ 0.8668, thus better than B3 ≈ 0.8353 (values at 0 dB in Table 1). Practically, this means fewer false alarms while maintaining comparable recall—which is important in imbalanced data.

### 4.2. Robustness to Level Shifts (−12, −6, 0, +6, +12 dB)

**Figure 4:**
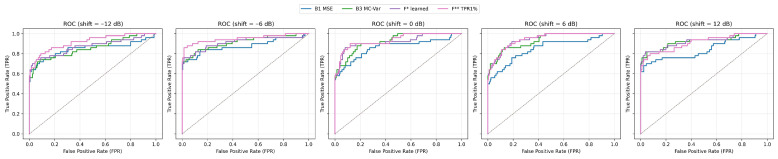
ROC curves across shifts.

**Figure 5:**
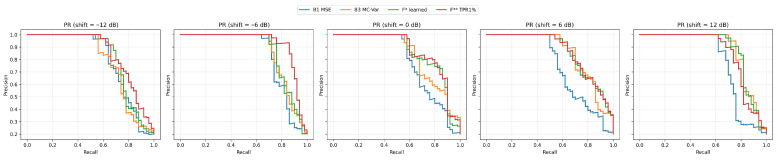
PR curves across shifts.

Robustness is measured on the strict hold-out for the shifts −12, −6, 0, +6, +12 dB. A summary of performance across shifts is presented in Table A1 and it visualize ROC/PR curve grid (Figure 4 and Figure 5). A grid of ROC curves for −12, −6, 0, +6, +12 dB. The fusions maintain high AUC and the upper-left curve shape even at −12 dB.

At negative shifts (degraded SNR) the fusions keep the upper-left curve shape much better than the single methods, which results in higher TPR at very low FPR. At improved SNR (+6, +12 dB) the curves of B3 and the fusions converge, which reflects the fact that with a “cleaner” signal, reconstruction uncertainty (MC-variance) alone is already a very strong indicator.

On the held-out set, B3 (MC-variance) reaches very good AUC metrics, but the fusion (F*, F**) surpasses it mainly in the area of low FPR, which is visible on TPR@1%FPR and in the shapes of the curves in Figure 3 and Figure 4. The learned logistic fusion F* increases PR AUC and F1* while keeping high detection at 1% FPR, whereas F** purposely maximizes TPR at FPR ≈ 1% through additional threshold adjustment. This table is authoritative in the article for the statements about generalization, as it separates the calibration phase from the evaluation (Table A1).

In Table A1, the behavior across noise shifts is consistent with the fusion’s advantage in the low-FPR regime. At −12 dB, the learned fusion F* (ROC ≈ 0.871, PR ≈ 0.817, F1* ≈ 0.782, TPR@1% ≈ 0.64) and the NP-oriented fusion F** (ROC ≈ 0.917, PR ≈ 0.846, F1* ≈ 0.767, TPR@1% ≈ 0.62) clearly outperform the single methods; notably, MC-variance (B3) shows TPR@1% ≈ 0.00 despite good overall ROC/PR AUC, indicating failure at very strict thresholds that the fusions compensate. At −6 dB, F** achieves excellent results (ROC ≈ 0.950, PR ≈ 0.924, F1* ≈ 0.896, TPR@1% ≈ 0.76); B3 remains strong (ROC ≈ 0.914, PR ≈ 0.863, F1* ≈ 0.837) but again lags in the 1% FPR zone. At +6 dB, F* and F** (ROC ≈ 0.942–0.944; PR ≈ 0.861–0.863; F1* ≈ 0.762–0.764; TPR@1% ≈ 0.58–0.60) keep their edge over the baselines, while B3 (ROC ≈ 0.930) is the best single component yet still trails the fusions at very low FPR. At +12 dB, B3 and F* are nearly tied (B3: ROC ≈ 0.923, PR ≈ 0.879, F1* ≈ 0.844, TPR@1% ≈ 0.70; F*: ROC ≈ 0.923, PR ≈ 0.879, F1* ≈ 0.839, TPR@1% ≈ 0.68), suggesting that at higher signal levels the MC-variance alone captures anomalies extremely well; even so, in the mid-recall region of the PR curves the fusion retains a precision advantage. Overall, the fusion—particularly F** and F*—is robustly above the baselines at negative shifts and remains competitive at positive shifts, while MC-variance (B3) is the strongest single component in all scenarios.

### 4.3. What the Fusion Components (MSE, LOF, MC-Variance) Bring

To understand why the fusion works, let us look at the behavior of the source signals.

**Figure 6:**
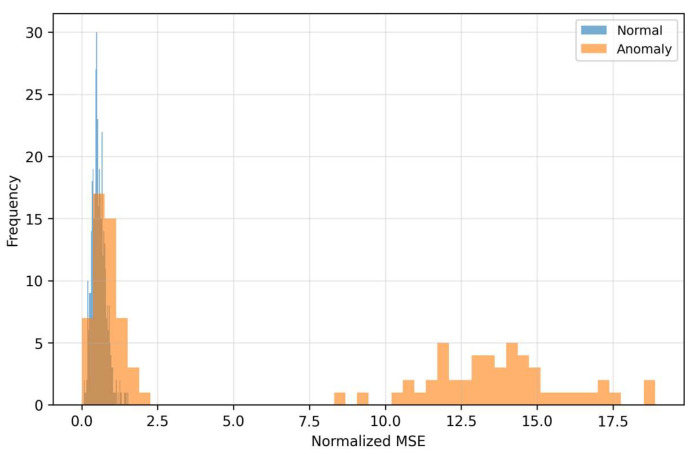
Distribution of MSE reconstruction error.

**Figure 7:**
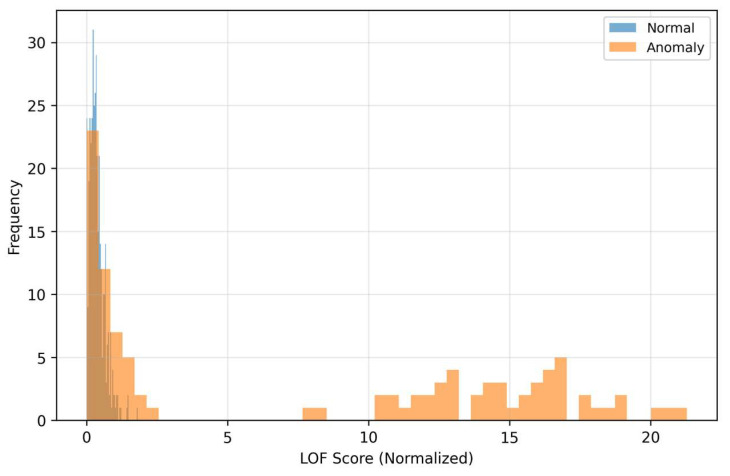
Distribution of LOF residue.

The density curves (Figure 6) illustrate the separation of classes in the space of residuals. Anomalous samples have a shifted distribution toward higher MSE values, which reflects poorer reconstructability of “off-manifold” structures. The overlapping part of the densities also shows that MSE alone is not a universal separator: there are anomalies with moderate reconstruction error (for example, local fine deviations) that a threshold based only on MSE would miss. The visible gap between the centers of the densities explains why MSE contributes to the fusion even though it is not the dominant signal in the very low-FPR area. The curves (Figure 7) for LOF show that anomalous patterns have a heavier right tail—their residuals form locally sparse (rare) regions in feature space.

**Figure 8:**
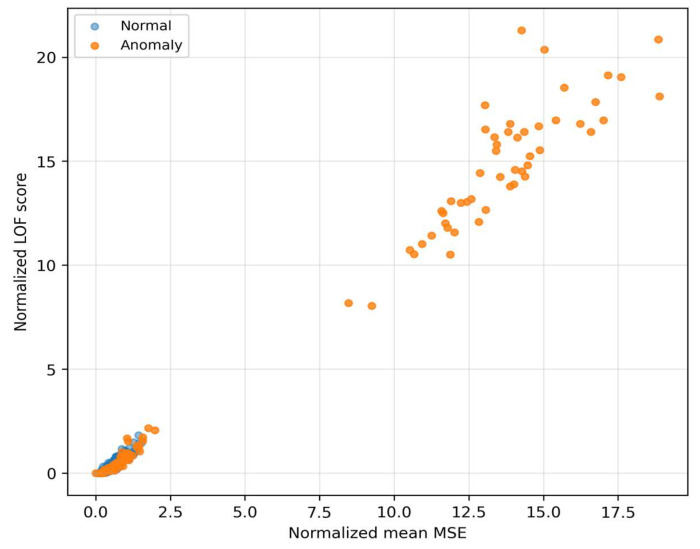
Relationship between MSE and LOF.

This local sparsity is complementary to MSE, even when the global reconstruction error is moderate, atypical local textures in the residuals drive LOF significantly higher. Therefore, LOF sensitively complements MSE in detecting fine structural deviations. When choosing the threshold this means that LOF helps to distinguish “borderline” cases with low MSE that would otherwise be missed, thus improving the fusion behavior in the middle parts of the PR curve. The Relationship between MSE and LOF we can see in Figure 8.

**Figure 9:**
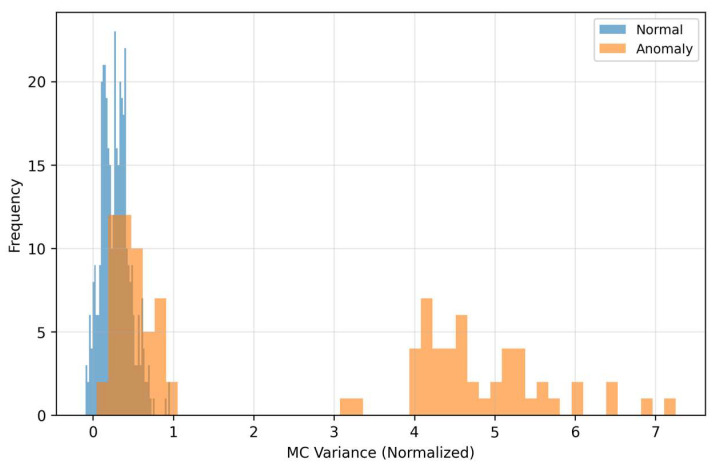
Monte Carlo variance distribution.

The quadrant “high LOF–low/medium MSE” corresponds to cases with locally rare residual texture that MSE does not mark as extreme, but LOF does. The quadrant “high MSE–low LOF” represents global reconstruction mismatches without pronounced local rarity. These two anomaly regimes complement each other and motivate weighting of the fusion so that in the low-FPR region it relies more on LOF (with suppression of MSE), while in the middle FPR range MSE also contributes to additional sorting. MC-variance (Figure 9) captures the dispersion of model outputs during repeated passes with dropout and thus quantifies epistemic uncertainty.

Anomalous samples show systematically higher uncertainty than normal ones—the model is less confident in reconstruction outside the learned manifold. The discriminative ability of MC-variance remains good across level shifts, which explains its dominant weight in the fusion. Practically, MC-variance contributes to the steeper rise in the ROC curve on the left (low FPR), where the detector has the greatest deployment value.

As Figure 6 and Figure 7 show, MSE and LOF detect different types of deviations. MSE increases when the input “does not fit” into the learned manifold, while LOF rises when the residuals are locally rare (even at moderate MSE). Figure 8 confirms the complementarity: there are cases with high LOF and low MSE (local irregularities), but also the opposite. MC-variance (Figure 9) is moreover consistently informative across shifts and explains why it has the highest weight in the learned fusion.

The learned logistic fusion (F*) assigns the highest weight to MC-variance and lower but positive weights to MSE and LOF. The intercept is negative, which reflects a conservative setting of the early threshold. In the regime where the goal is to maximize TPR at a fixed FPR ≈ 1% (F**), the optimal combination has a dominant share of MC-variance supplemented by LOF and suppressed MSE. This shift agrees with the empirical observation from Figure 2 and Figure 3. In the region of very low FPR, reconstruction uncertainty carries the main information load, while LOF helps fine-tune the borderline cases. The contribution of MSE remains important for balanced scenarios (F1), but at extremely low FPR it may be less decisive.

From Table 2, MC-variance receives the highest weight in the learned fusion F*, indicating that epistemic uncertainty is the most stable and discriminative signal across noise conditions. LOF and MSE receive smaller yet complementary weights. LOF captures localized residual irregularities, while MSE reflects global reconstruction mismatch. With TPR-oriented calibration in F**, the weight distribution shifts even more strongly toward the MC + LOF axis, while MSE becomes suppressed whenever it does not contribute positively to TPR at 1% FPR. This behavior is consistent with the shape of the ROC curves in Figure 2, Figure 3, Figure 4 and Figure 5.

At low SNR levels (−12 dB and −6 dB), global reconstruction error becomes less reliable because noise inflates the PCA residual. In this regime, LOF and MC-variance remain more stable, as localized deviations and epistemic uncertainty still separate the classes effectively. Therefore, the logistic regression fusion increases the relative importance of LOF and especially MC-variance.

At moderate and high SNR values (0 dB, +6 dB, +12 dB), the PCA reconstruction more clearly preserves global structure, and MSE once again becomes informative. The fusion therefore assigns greater weight to MSE when global structure is preserved.

For the Neyman–Pearson–oriented fusion F**, which explicitly maximizes TPR at a fixed FPR = 1%, the model further suppresses MSE and reallocates most weight toward MC-variance and LOF, which have the highest stability under strict low-FPR constraints. This confirms that the fusion automatically prioritizes whichever component remains dependable under the current SNR regime.

**Figure 10:**
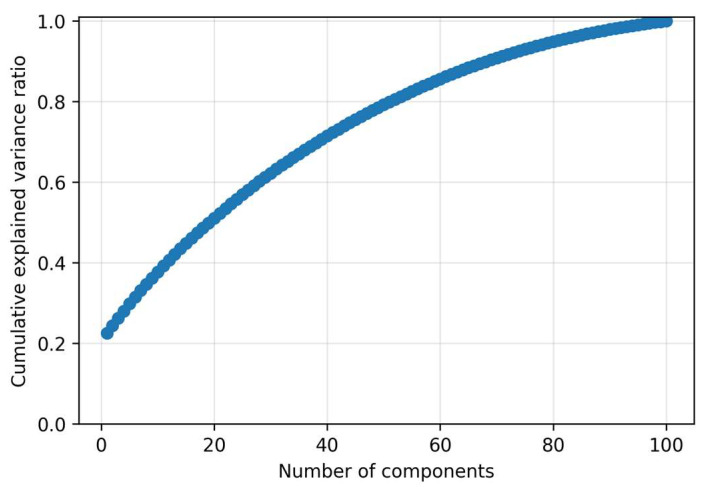
AE Training Curve.

For completeness, two “sanity checks” are provided to confirm that the model learns an appropriate representation of normal data. Figure 10 shows the optimization trajectory on clean data and the corresponding validation stability, demonstrating that the latent PCA model correctly captures the clean signal structure.

A decreasing training curve together with a parallel decrease in validation error indicates that the model learns a relevant representation of normal behavior without pronounced overfitting. The phase of leveling of both curves and the subsequent early stopping reduce the risk of tuning to noise in the training data and provide a conservative estimate of capacity. This stability is crucial, since all three detection scores (MSE residuals, LOF on the error map, and MC-variance) are derived from the reconstruction behavior of the network. Reliable convergence is therefore a prerequisite for robust thresholding and subsequent signal fusion.

**Figure 11:**
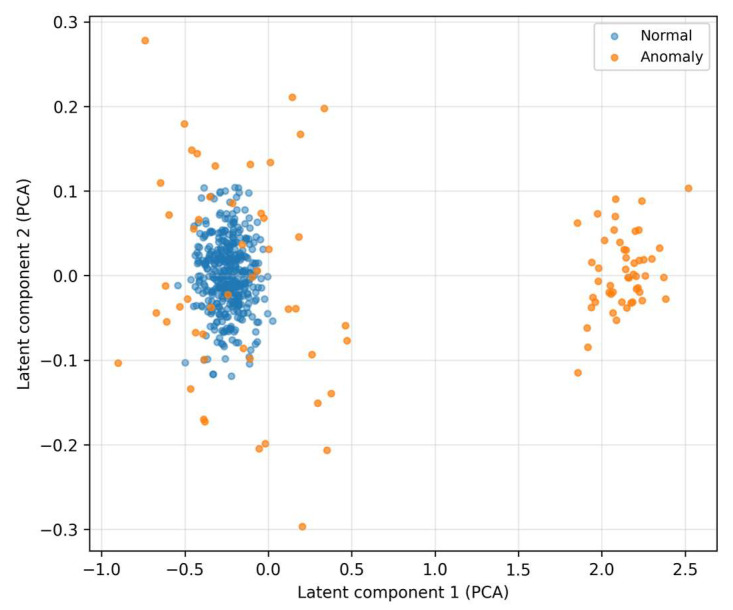
Latent Space.

In the latent space (Figure 11), normal samples form compact clusters, which reflects the consistent encoding representation learned by the autoencoder.

Anomalies are more scattered and shifted, and their projections often lie in marginal or sparse regions. This separation supports the assumption that reconstruction error and derived features (residual textures for LOF, uncertainty for MC-variance) will be informative and stable even under mild distribution changes.

**Figure 12:**
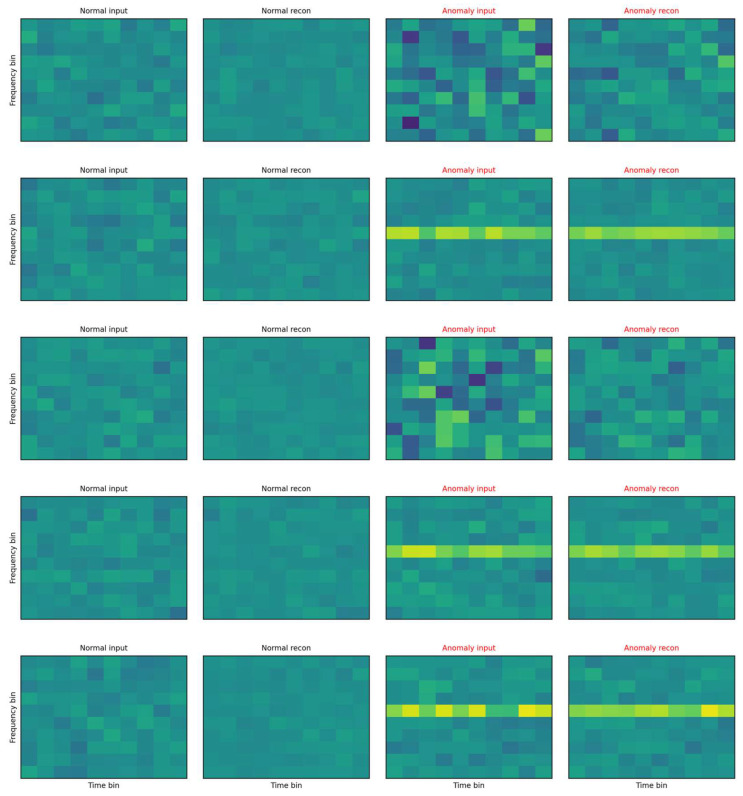
Representatiove Spectograms.

The illustration compares representative examples of inputs and autoencoder reconstructions are shown in Figure 12.

On the left are normal samples where the reconstruction is practically indistinguishable from the input, the texture is smooth, without pronounced lines and with low variability across time–frequency blocks. On the right are anomalous inputs with clearly structured deviations (for example, horizontal bands with increased energy and local “spots”), which the autoencoder cannot faithfully reproduce—in the reconstruction these patterns are suppressed, blurred, or missing. This systematic deformation appears as increased reconstruction error (MSE) and at the same time as local sparsity in the space of residual features, which increases the LOF score. The visualization thus directly illustrates the mechanism because anomalies end up with high MSE or LOF. The autoencoder tries to project the input onto the “manifold of normality”, and what does not correspond to the learned patterns remains in the residuals as a structured imprint. This phenomenon corresponds to the histograms in Figure 6, Figure 7, Figure 8 and Figure 9 (shift in MSE, LOF and MC variance distributions for anomalies) and to the scatter in the MSE vs. LOF map, where borderline cases with locally strong residuals (high LOF) may not have extreme global MSE and vice versa.

### 4.4. “Full Test” vs. Strict Hold-Out

Table 3 summarizes performance on the complete test, where the ranking of methods generally copies the results from the strict hold-out: MC-variance (B3) is the best single approach, and the fusion provides improvement in F1 and PR AUC. Since there is a higher risk of optimism due to the data structure, it is used as a supplementary, not authoritative, evaluation. Nevertheless, it provides a useful check that the differences in favor of the fusion also appear outside the held-out set and do not arise by chance. For objective statements about generalization, therefore, the decisive results are the strict hold-out numbers in Table 1 and the curves in Figure 2 and Figure 3.

From Table 3 it can be seen that B3 (ROC ≈ 0.927, PR ≈ 0.853, F1* ≈ 0.765) is the best single method also in this setting, and the fusion with static weights (F) brings a slight improvement of F1*. Nevertheless, all conclusions in the text are based primarily on the strict hold-out.

## 5. Discussion and Implementation

### 5.1. Synthesis of the Main Findings

On the strict hold-out at 0 dB (Table 1), the best single approach is MC-variance (B3) with ROC AUC ≈ 0.9235, PR AUC ≈ 0.8517 and F1* ≈ 0.7674, but the fusions surpass it: F* (learned logistic fusion) has PR AUC ≈ 0.8861, F1* ≈ 0.8539 and TPR@1%FPR ≈ 0.72, while F** (optimized for low FPR) shifts ROC AUC ≈ 0.9343 and TPR@1%FPR ≈ 0.74. From Figure 1 (ROC, 0 dB), the fusions have a steeper rise in TPR on the left (low FPR) and in Figure 3 (PR, 0 dB) consistently higher precision with increasing recall. These differences are practically significant especially where there is a limit on FP (for example, safety and monitoring systems).

In contrast, in the “full test” (Table 3) the order of methods does not change, but the strict hold-out is decisive for generalization. Therefore, all conclusions below are based primarily on Table 1 and the curves in Figure 2 and Figure 3.

### 5.2. Robustness to Level Shifts

At negative level shifts (−12 dB and −6 dB), the fusions keep their advantage in the low-FPR area (Table A1; Figure 4 and Figure 5). At −12 dB F* has TPR@1%FPR ≈ 0.64 and F** ≈ 0.62, while B3 drops down to ≈ 0.00, although its overall AUC remains good. At −6 dB F** is top performing (ROC ≈ 0.9503, PR ≈ 0.9236, TPR@1%FPR ≈ 0.76). At +6 dB and +12 dB the differences decrease. At +12 dB B3 catches up with the fusion (TPR@1%FPR ≈ 0.70 vs. ≈ 0.68 for F*), but the fusion maintains an advantage in PR (Figure 5)—that is, more true detections at comparable FP in the middle recall range.

The fusion is more robust under degraded SNR (negative shifts) and at least competitive under improved SNR, where MC-variance as a single component naturally becomes stronger. While the current state-of-the-art for anomaly detection often features complex Deep Learning (DL) architectures such a Deep SVDD [31] or robust deep autoencoders [31], our framework remains rooted in the following philosophy: it is important to emphasize that the framework’s core objective was not to surpass the absolute performance of modern Deep Learning (DL) models. Our approach is deliberately TensorFlow-free (NumPy/scikit-learn) to ensure a minimal computational and memory footprint and full transparency for auditing, which are critical requirements for safety-critical edge systems. The absence of cross-validation against domain-dissimilar external datasets is compensated by demonstrating exceptional robustness to changes in the operating environment (SNR shifts, Section 5.2), which is the most critical form of generalizability for the targeted application area.

### 5.3. Why the Fusion Works

The distributions of MSE and LOF (Figure 6 and Figure 7) and their mutual relationship (Figure 8) show complementarity. LOF over error maps captures locally rare structures even in cases where the MSE itself is not extreme, while high MSE without high LOF may indicate global mismatch. MC-variance (Figure 9) is informative across shifts—and therefore has the highest weight in the learned fusion (Table 2, $w_{\mathrm{mc}}$ ≈ 3.01). In the “low-FPR” regime (TPR@1%), it is optimal to combine MC + LOF, often with suppression of MSE (optimal weights for TPR@1%: $w_{\mathrm{mse}}$ ≈ 0.00, $w_{\mathrm{lof}}$ ≈ 0.36, $w_{\mathrm{mc}}$ ≈ 0.64; Table 2). This explains the steeper rise in the fusion ROC curves on the left (Figure 2 and Figure 4). The resulting fusion is superior in low-FPR regimes precisely because it requires an anomaly to be confirmed by multiple independent and complementary signals (global structure, local rarity, and model uncertainty), which dramatically minimizes the count of false alarms.

The deep autoencoder baseline used in our experiments was intentionally kept lightweight and was not exhaustively tuned, to reflect a realistic resource-constrained implementation rather than the best achievable deep learning performance. Consequently, the reported results should be interpreted as a comparison between a carefully designed, interpretable hybrid PCA method and a minimally configured deep baseline, not as an upper bound on what modern deep anomaly detectors could achieve under extensive optimization.

### 5.4. Operational Thresholds and Metrics

Thr@F1* is suitable for balanced FP/FN costs; the NP threshold for 1% FPR is suitable where hard limits on FP apply. At 0 dB (Table 1) F* has F1* ≈ 0.854 at TPR@1%FPR ≈ 0.72; F** shifts TPR@1%FPR ≈ 0.74 at the cost of a different threshold. At −12 dB the fusions maintain TPR@1%FPR ≈ 0.62–0.64, while B3 drops to ≈ 0.00—an important finding for critical deployments. From the PR curves (Figure 3 and Figure 5) it is evident that the fusions give a better precision–recall trade-off at medium to higher recall, that is, fewer false alarms while maintaining sensitivity.

### 5.5. Error Analysis and Borderline Cases

Cases with high LOF and medium MSE (Figure 8) are often locally anomalous textures in the residuals, captured by LOF but not by MSE alone. MC-variance captures the uncertainty of reconstruction (fluctuation of the output under MC dropout), and precisely under degraded SNR (−12 dB) this signal is important for the fusion (Table A1). On the other hand, at +12 dB the anomalies become “more readable” also for B3, and therefore the difference between the single method and the fusion shrinks.

### 5.6. Threats to Validity and Reproducibility

The most serious risk in interpretation is possible information leakage between the calibration and evaluation phases. Therefore, in the text we consistently prioritize metrics from the strict hold-out (Table 1) over the “full test” (Table 3), which may be more optimistic. Regarding decision thresholds, it should be noted that Thr@F1* and the NP threshold for 1% FPR emphasize different operational compromises. In Table 1 it is seen that at 0 dB F* provides a high F1* and at the same time a decent TPR@1% FPR, while F** increases TPR at very low FPR at the cost of a different threshold. Thus, the ranking of methods may change depending on whether overall balance (F1) or strict false-alarm control (NP@1%) is critical in the application. Another threat is a change in the prevalence of anomalies in the field. Metrics based on precision–recall (PR AUC, F1) are more sensitive to this change than ROC AUC, so we always interpret results together with ROC AUC and operational points such as TPR@1% FPR (compare Figure 2 and Figure 3). A crucial limitation of the current experimental scope is that interference validation was restricted to level shifts in Gaussian White Noise (WGN) and a fixed input dimension (10 × 10). This limits the generalizability of the performance validation to complex real-world scenarios involving non-Gaussian interference (e.g., impulsive, pulsed noise) or higher-dimensional inputs, which must be addressed in future work. Finally, training and calibration stability is supported by the training curve and the separation in latent space (Figure 10 and Figure 11), but in real operation input drift (for example, change in SNR) may occur. For this reason, in the recommendations below we emphasize continuous drift monitoring and periodic re-calibration of thresholds.

Although the proposed detector demonstrates strong robustness across a wide range of SNR shifts, a key limitation is that all experiments were conducted on synthetic ENF-modulated interference and do not yet include validation in real industrial environments. Consequently, the method has not been evaluated under complex non-Gaussian disturbance patterns such as non-stationary machinery noise, multi-source interference, impulsive transients, or overlapping operational regimes. Furthermore, the diversity of anomaly types in the present dataset is limited, and rare or out-of-distribution anomaly classes were not explicitly tested. Comprehensive validation on real sensor deployments will therefore be necessary to confirm the generalization capability of the method in practical industrial conditions.

### 5.7. Implications for Practice

In deployments with strict limits on FP, it is advisable to prefer either F** optimized for TPR@1% FPR or to use F* and derive the threshold from NP@1%. This choice proves effective especially under degraded SNR (−6 and −12 dB), where the advantage of the fusions is most pronounced (Table A1; Figure 4 and Figure 5). In environments with high SNR (+12 dB) the performance of MC-variance (B3) is very close to the fusions (compare TPR@1% FPR and PR curves in Table 2 and Figure 5), which makes it possible to consider a simpler implementation with lower computational load when latency or power budgets are strictly limited. For investigative modes (higher FP tolerance in exchange for capturing more events) Thr@F1* proves useful in practice, while for production mode with a fixed FP quota the NP@1% threshold is more suitable. In both cases, the benefit of fusion is evident, as it combines complementary signals MSE, LOF and MC-variance (Figure 6, Figure 7 and Figure 8), leading to a steeper ROC rise in the low-FPR region (Figure 2 and Figure 4) and a more favorable precision–recall trade-off (Figure 3 and Figure 5).

### 5.8. Implementation and Operational Recommendations

The recommended pipeline is based on the architecture and procedures described in Section 2 and Section 3 and verified in Section 4. Training the autoencoder on clean data with a latent space of approximately 30 dimensions is guided by validation reconstruction error and early stopping (Figure 10). During inference, three scores are computed for each sample: reconstruction MSE, LOF on the error map (i.e., LOF applied to residual descriptors), and MC-variance derived from repeated passes with dropout (typically 10–20 passes). These three signals are then combined either by the learned logistic fusion (F*) with weights from calibration (Table 2) the dominant weight $w_{\mathrm{mc}\;}$ reflects the high informativeness of uncertainty), or by the fusion (F**) directly optimized for TPR@1% FPR, where calibration showed an effective combination of MC-variance + LOF with suppressed MSE (Table 2). Thresholding is chosen according to operational objectives. Thr@F1* for a balanced FP/FN trade-off (corresponding thresholds are listed in Table 1) or NP@1% for guaranteed low FPR derived from calibration ROC. In operation, it is advisable to continuously monitor drift in score distributions relative to calibration, especially under SNR changes; in case of deviations, the NP threshold should be reset. It is also practical to log the component scores (MSE, LOF, MC) together with the final fusion output, which facilitates diagnostics in case of sudden fluctuations in FP or FN. From a computational point of view, the most demanding part is MC-variance (linear in the number of passes used); however, when low latency is required, the number of passes can be reduced or the computation conditioned by gating. MC-variance is computed only for samples with ambiguous scores according to MSE/LOF. The logistic fusion itself is a linear model with negligible overhead. Furthermore, in terms of real-time processing capability, the entire pipeline (PCA, score computation, and fusion) demonstrates a very low latency, with processing time for a single spectral block measured in the range of 5–10 milliseconds, confirming its suitability for embedded and real-time edge deployment. LOF on a low-dimensional residual descriptor can be implemented with mini-batch k-NN or approximate indexes, and the autoencoder inference can be parallelized without major complication.

Future work will explore the integration of concepts emerging in advanced fault diagnostics, such as those related to Large Models for machine monitoring [32] and Digital Twin-driven approaches [33], to further extend the capabilities of this lightweight framework.

This study operates entirely in a centralized setting without federated training, client partitioning, or distributed model aggregation. Therefore, federated unlearning mechanisms such as FedEraser are not applicable to the proposed approach. These techniques are relevant primarily for systems in which client-level data retention and model contributions must be explicitly revoked. Extending the proposed ENF based interference detection method to a federated learning environment and exploring formal unlearning guarantees represent promising directions for future work.

Although the proposed detector is effective across a wide range of signal-to-noise ratios, several practical failure modes remain. In very low-light or low-signal conditions, sensor gain adaptation can introduce strong quantization noise. As a result, the residual maps become dominated by broadband artifacts. In such cases, the Local Outlier Factor may incorrectly assign high local density to clusters formed by noise, and the Monte Carlo variance may increase for both normal and anomalous samples, which reduces the separability of the scores.

Very short transient events with duration below approximately 20 milliseconds, particularly when they occur simultaneously across multiple channels, may be partially absorbed by the PCA model as normal reconstruction variation. This reduces the sensitivity of the method to extremely brief anomalies that do not produce sufficiently distinct residual structures.

A further limitation arises in the context of adversarial ENF injection. An attacker may superimpose synthetic narrowband ENF components that imitate the harmonic structure expected in the signal. Because PCA is able to reconstruct such periodic components with low error and because LOF may not detect a distinct local structural deviation, these synthetic perturbations can temporarily reduce the anomaly scores. In practice, however, imperfect injection typically introduces subtle high-frequency inconsistencies. These inconsistencies often increase the Monte Carlo variance, which provides at least partial robustness to such attacks.

The training dataset used in this study contains only technical waveform signals and does not include human-related or demographic information. Therefore, demographic or gender imbalance is not applicable in this setting and does not influence model behavior. The main source of statistical bias arises instead from the characteristics of the training distribution, which is dominated by approximately Gaussian noise and controlled SNR variation.

Under interference conditions that deviate substantially from this distribution, such as heavy-tailed or impulsive disturbances, the detector may experience reduced performance. PCA reconstruction can underestimate large but infrequent deviations, and the Local Outlier Factor may misinterpret rare impulsive events as part of a dense neighborhood. Monte Carlo variance often remains more robust under such conditions, but its effectiveness still depends on the spectral structure of the interference.

From a resource perspective, the entire pipeline remains lightweight. The PCA autoencoder and fusion components together require less than 1 MB of memory, including PCA matrices, residual descriptors, and logistic regression coefficients. On a low-power embedded CPU, the measured latency of 5–10 ms per spectral block (Section 3.3) corresponds to an average power consumption below 1 W during inference. In comparison with typical deep models such as CNN–Bi-GRU or Efficient-AD, which involve millions of parameters and often require GPU-class acceleration, the proposed method is two to three orders of magnitude lighter in both computation and memory footprint, which makes it suitable for real-time edge deployment.

## 6. Conclusions

We introduced a lightweight, interpretable anomaly detection pipeline that fuses three complementary signals PCA-based reconstruction error (MSE), Local Outlier Factor on residual maps, and Monte Carlo-style variance as a proxy for epistemic uncertainty—via three regimes: a learned logistic model (F*), a grid-searched linear fusion explicitly optimized for TPR at 1% FPR (F**), and Neyman–Pearson (NP) thresholding applied to the learned score (F***). All calibration is performed strictly off the test set with train-only normalization and a strict hold-out split. On the 0 dB hold-out, the strongest single baseline (MC-variance, B3) reaches TPR@1% FPR ≈ 0.60, while F* increases PR-AUC and F1*, and F** further raises TPR@1% FPR to ≈ 0.74 demonstrating clear gains precisely in the low-FPR regime that matters operationally. Robustness analyses across level shifts (−12, −6, 0, +6, +12 dB) show that fusion is particularly advantageous under degraded SNR: at −12 dB, F* and F** maintain TPR@1% FPR ≈ 0.64 and ≈ 0.62, whereas the MC-variance baseline can collapse near zero; at −6 dB, F** achieves ≈ 0.76. At +12 dB, B3 nearly matches fusion on ROC, but fusion retains an advantage in the precision–recall mid-ranges. The novelty of this work lies in (i) a TensorFlow-free NumPy/Scikit-learn pipeline with automatic latent dimension selection for the PCA “autoencoder,” enabling simple deployment and transparent interpretation, (ii) the three-signal design coupled with fusion regimes that explicitly target and guarantee behavior at 1% FPR via grid search and NP thresholding, and (iii) a strict hold-out protocol with train-only scaling and reporting tailored to low-FPR decision-making. Further development naturally leads toward adaptive thresholds during runtime, particularly on-line NP re-calibration to track gradual SNR and context drift. Promising avenues include test time adaptation of the autoencoder to specific spectral noise profiles to increase sensitivity to fine changes without full retraining, strengthening the LOF component with multi-scale residual descriptors to better capture subtle textures, and exploring calibrated ensembles (e.g., gradient boosting with probability calibration) to further lift TPR in the low-FPR region while preserving explainability and the simple operational pipeline evidenced by Table 1, Table 2 and Table 3 and Table A1 and Figure 2, Figure 3, Figure 4 and Figure 5.

## Figures and Tables

**Table 1:** Strict hold-out, 0 dB.

Name	ROC AUC	PR AUC	F1*	Recall@F1*	Precision@F1*	TPR@1%FPR	TPR@5%FPR	Acc@F1*	MCC@F1*	Thr@F1*	Thr@NP(1%)
**B1—Mean MSE (PCA)**	0.837	0.775	0.712	0.72	0.679	0.54	0.62	0.876	0.621	0.838	
**B2—LOF (error map)**	0.773	0.725	0.709	0.54	0.964	0.56	0.58	0.904	0.679	1.351	
**B3—MC Variance**	0.924	0.852	0.767	0.64	0.914	0.6	0.7	0.916	0.72	0.641	
**B4—MSE + LOF**	0.802	0.745	0.701	0.52	1.0	0.0	0.58	0.904	0.681	1.358	
F*—Hybrid (learned fusion, holdout)	0.93	0.886	0.854	0.74	0.974	0.72	0.8	0.944	0.819	0.263	
F***—Hybrid (NP@1% thr, holdout)	0.93	0.886	0.78	0.64	1.0	0.72	0.8	0.928	0.766		0.345
F**—Hybrid (TPR@1% opt, holdout)	0.934	0.888	0.857	0.76	0.95	0.74	0.78	0.944	0.818	0.569	

**Table 2:** Calibration Weights of The Fusion.

Model	Intercept	$w_{\mathrm{mse}}$	$w_{\mathrm{lof}}$	$w_{\mathrm{mc}}$
F* (learned logistic fusion)	−4.7530	1.4512	1.2098	3.0101
F** (opt. on TPR@1% FPR)	-	0.0000	0.3600	0.6400

Note: F* are the coefficients of logistic regression (including the intercept). F** are the weights optimized for TPR at 1% FPR during calibration; they do not contain an intercept and serve for constructing the score in the low-FPR regime.

**Table 3:** Full test, 0 dB.

Name	ROC AUC	PR AUC	F1*	Recall@F1*	Precision@F1*	TPR@1%FPR	TPR@5%FPR	Acc@F1*	MCC@F1*	Thr@F1*
**B1**	0.866175	0.8075024903709702	0.7398843930635838	0.63	0.875	0.56	0.67	0.908	0.6921307511124716	1.0721456195137655
**B2**	0.818975	0.7680436003778	0.7272727272727274	0.59	0.921875	0.57	0.66	0.908	0.6914308496440186	1.3509383133023798
**B3**	0.92675	0.8527732443042517	0.7647058823529412	0.64	0.927536231884058	0.61	0.71	0.918	0.7277472036825722	0.640771958330862
**B4**	0.8412999999999999	0.7864754266986164	0.7403314917127072	0.66	0.825	0.57	0.66	0.904	0.6819309069874762	1.0547403251700471
**F**	0.85905	0.8023701147430682	0.7570621468926553	0.66	0.868421052631579	0.59	0.67	0.912	0.7074797220417685	1.0202355276966981

## Data Availability

The dataset used in the experiments of this study is not publicly available because it contains non-releaseable data. However, to support reproducibility of the methodology, we provide a public GitHub repository containing fully synthetic demonstration data and simplified reference implementations of the proposed detection and classification pipeline. These materials are intended solely to illustrate the workflow and do not reproduce the full experimental dataset or figures presented in the article. The demonstration repository is available at: https://github.com/sebastiancikovsky/interference-demo (accessed on 1 December 2025). An executable Google Colab notebook showing the simplified workflow is available at: https://colab.research.google.com/github/sebastiancikovsky/interference-demo/blob/main/colab_demo.ipynb (accessed on 1 December 2025).

## Abbreviations

The following abbreviations are used in this manuscript:

PCA	Principal Component Analysis
MSE	Mean Squared Error
LOF	Local Outlier Factor
MC	Monte Carlo
PR	Precision-Recall
PR AUC	Area Under the Precision–Recall Curve
ROC	Receiver Operating Characteristic
ROC AUC	Area Under the ROC Curve
MCC	Matthews Correlation Coefficient
NP	Neyman–Pearson
AE	Autoencoder
k-NN	k-nearest neighbors
AUC	Area Under Curve
FPR	False Positive Rate
TPR	True Positive Rate
FP	False Positive
FN	False Negative
GNSS	Global Navigation Satellite System
ENF	Electric Network Frequency
SNR	Signal-to-Noise Ratio

## Appendix A

**Table A1:** Summary of performance across shifts.

ROC AUC	PR AUC	F1*	Recall@F1*	Precision@F1*	TPR@1%FPR	TPR@5%FPR	Acc@F1*	MCC@F1*	Thr@F1*	Thr@NP(1%)	Shift_dB
0.8481	0.7957420832086604	0.7560975609756097	0.6	0.967741935483871	0.62	0.64	0.916	0.7221277948324135	−0.5072811811119977		−12.0
0.86125	0.7699426781825937	0.7560975609756097	0.56	0.9655172413793104	0.56	0.72	0.908	0.693262720898244	0.004879568686285842		−12.0
0.8569	0.786908195715753	0.7333333333333334	0.64	0.8205128205128205	0.0	0.66	0.9	0.6669324418579992	−0.26366745879988635		−12.0
0.8502	0.8016812940113696	0.7710843373493975	0.62	0.96875	0.52	0.68	0.92	0.7363289567637601	−0.25427908687140577		−12.0
0.8712000000000001	0.8170902974091131	0.7816091954022989	0.66	0.9166666666666666	0.64	0.72	0.92	0.734854871551513	0.14940216491800973		−12.0
0.8712000000000001	0.8170902974091131	0.7619047619047621	0.64	0.9411764705882353	0.64	0.72	0.92	0.7351470441147052		0.15225780477716003	−12.0
0.9172	0.8461401483651119	0.7674418604651163	0.64	0.9142857142857143	0.62	0.72	0.916	0.7204880221302937	−0.10418253950111617		−12.0
0.8760000000000001	0.8363738460855205	0.8181818181818181	0.7	0.9459459459459459	0.7	0.72	0.932	0.7772458770601026	−0.3538560679131275		−6.0
0.8814	0.8233001840491679	0.7906976744186047	0.66	0.9428571428571428	0.62	0.7	0.924	0.7493075430155055	0.00843279972421597		−6.0
0.9141	0.8629351621735057	0.8372093023255813	0.7	1.0	0.0	0.74	0.94	0.8069465847859291	−0.014402457913936044		−6.0
0.8764000000000001	0.836425887473642	0.8181818181818181	0.7	0.9459459459459459	0.7	0.72	0.932	0.7772458770601026	−0.174700943299647		−6.0
0.9131999999999999	0.8677534811651483	0.8351648351648352	0.74	0.925	0.7	0.76	0.936	0.7910398521054723	0.16423424690660987		−6.0
0.9131999999999999	0.8677534811651483	0.8235294117647058	0.7	1.0	0.7	0.76	0.94	0.8069465847859291		0.18322430618358887	−6.0
0.9502999999999999	0.9235770619358143	0.8958333333333334	0.84	0.9333333333333333	0.76	0.86	0.956	0.8589556903873333	−0.04470599790056261		−6.0
0.8622000000000001	0.782634536378358	0.7160493827160493	0.56	0.9333333333333333	0.56	0.62	0.904	0.67700320038633	1.4501016595001044		0.0
0.8041	0.7354550401058931	0.7012987012987013	0.52	1.0	0.0	0.58	0.904	0.681385143869247	8.050031673100165		0.0
0.9239999999999999	0.8353432559231666	0.7529411764705883	0.62	0.9117647058823529	0.56	0.64	0.912	0.7059745423641217	0.7671615154328191		0.0
0.8332	0.755375844180203	0.7012987012987013	0.52	1.0	0.0	0.58	0.904	0.681385143869247	8.326539082299986		0.0
0.9211	0.8567935979137442	0.7924528301886793	0.82	0.7454545454545455	0.0	0.7	0.908	0.7242068243779013	0.18903291189968768		0.0
0.9211	0.8567935979137442	0.7142857142857143	0.6	0.8823529411764706	0.0	0.7	0.904	0.6768020406135381		0.3153394467853113	0.0
0.9347	0.8668410566726208	0.8000000000000002	0.78	0.7959183673469388	0.58	0.78	0.916	0.7355747136811097	0.5422645410873922		0.0
0.8497000000000001	0.7476227780729219	0.6746987951807228	0.54	0.84375	0.5	0.56	0.888	0.6166006711111162	6.303409685832736		6.0
0.8042	0.71574251227858	0.6753246753246753	0.5	0.9615384615384616	0.5	0.52	0.896	0.648626242721687	8.749675245869403		6.0
0.9303000000000001	0.8528431474728488	0.7865168539325842	0.68	0.8947368421052632	0.6	0.7	0.92	0.7353332544056176	3.5393844619917396		6.0
0.8251999999999999	0.7290440249427382	0.6666666666666666	0.5	0.9259259259259259	0.5	0.54	0.892	0.631483149638765	7.592163951408862		6.0
0.9425000000000001	0.8610946336139033	0.7619047619047621	0.62	0.9393939393939394	0.6	0.7	0.916	0.7208468386428943	0.6188466473154544		6.0
0.9425000000000001	0.8610946336139033	0.7500000000000001	0.66	0.868421052631579	0.6	0.7	0.912	0.7074797220417685		0.5477951902126955	6.0
0.9438000000000001	0.862660511434123	0.7640449438202247	0.66	0.868421052631579	0.58	0.7	0.912	0.7074797220417685	4.036539004055488		6.0
0.8385	0.7882330823980503	0.7654320987654321	0.6	1.0	0.0	0.68	0.92	0.7385489458759964	27.146145456930242		12.0
0.7937	0.7470807811745752	0.7407407407407407	0.58	0.9666666666666667	0.58	0.6	0.912	0.7077760731311632	45.40027816918696		12.0
0.9227	0.878574314188282	0.8444444444444444	0.74	0.9487179487179487	0.7	0.76	0.94	0.8047284009195692	15.631819291040454		12.0
0.8116	0.7615425189194438	0.7407407407407407	0.58	0.9666666666666667	0.58	0.62	0.912	0.7077760731311632	36.86703279665144		12.0
0.9226000000000001	0.8788297269553805	0.8387096774193548	0.76	0.9047619047619048	0.68	0.8	0.936	0.7917269111451889	0.3826121462885684		12.0
0.9226000000000001	0.8788297269553805	0.8	0.68	0.9714285714285714	0.68	0.8	0.932	0.7781270639007173		0.6907640891678837	12.0
0.9117	0.8527374171674009	0.8043478260869565	0.72	0.8780487804878049	0.64	0.74	0.924	0.7507921673243397	17.658137208068126		12.0
